# Pharmacological Efficacy and Gastrointestinal Safety of Different Aspirin Formulations for Cardiovascular Prevention: A Narrative Review

**DOI:** 10.3390/jcdd10040137

**Published:** 2023-03-23

**Authors:** Bianca Clerici, Marco Cattaneo

**Affiliations:** 1 Divisione di Medicina Generale II, ASST Santi Paolo e Carlo, Dipartimento di Scienze della Salute, Università degli Studi di Milano, Via Di Rudinì 8, 20142 Milano, Italy; 2 Fondazione Arianna Anticoagulazione, Via Paolo Fabbri 1/3, 40138 Bologna, Italy

**Keywords:** aspirin, coronary artery disease, cerebrovascular disease, diabetes mellitus, essential thrombocythemia, platelet function, thromboxane, gastrointestinal bleeding, enteric-coated aspirin, cardiovascular prevention

## Abstract

Aspirin inhibits platelet function by irreversibly inhibiting the synthesis of thromboxane A2 (TxA2). Aspirin, at low doses, is widely used for cardiovascular prevention. Gastrointestinal discomfort, mucosal erosions/ulcerations and bleeding are frequent complications of chronic treatment. To reduce these adverse effects, different formulations of aspirin have been developed, including enteric-coated (EC) aspirin, the most widely used aspirin formulation. However, EC aspirin is less effective than plain aspirin in inhibiting TxA2 production, especially in subjects with high body weight. The inadequate pharmacological efficacy of EC aspirin is mirrored by lower protection from cardiovascular events in subjects weighing >70 kg. Endoscopic studies showed that EC aspirin causes fewer erosions of the gastric mucosa compared to plain aspirin (which is absorbed in the stomach) but causes mucosal erosions in the small intestine, where it is absorbed. Several studies demonstrated that EC aspirin does not reduce the incidence of clinically relevant gastrointestinal ulceration and bleeding. Similar results were found for buffered aspirin. Although interesting, the results of experiments on the phospholipid-aspirin complex PL2200 are still preliminary. Considering its favorable pharmacological profile, plain aspirin should be the preferred formulation to be used for cardiovascular prevention.

## 1. Background

Acetylsalicylic acid, the active principle of aspirin, irreversibly inhibits the activity of platelet cyclo-oxygenase-1 (COX-1), thereby inhibiting the platelet production of the pro-aggregatory and vasoconstrictor molecule thromboxane A2 (TxA2) [1,2]. Due to its inhibitory effect on platelet function, aspirin is widely used as an antithrombotic drug for the treatment of acute coronary syndromes and cerebrovascular accidents and for their secondary prevention; its role in the primary prevention of these disorders is less well established [3]. A common complication of chronic treatment with aspirin is the increased risk of gastrointestinal (GI) discomfort, mucosal erosions/ulcerations and bleeding, which are frequently observed despite the fact that prevention of thrombosis can be obtained by administering low-dose aspirin (75–100 mg o.d.) [2]. A placebo-controlled study showed that the incidence of bleeding peptic ulcers in subjects on cardiovascular prophylaxis with low-dose aspirin was 40–80% higher than in placebo-treated subjects [4], while a Danish cohort study of 27,694 individuals showed that the standardized incidence rate ratio of upper GI bleeding (UGIB) was 2.6 among users of low-dose aspirin [5]. A meta-analysis of 24 randomized clinical trials (RCTs) on the risk of GI hemorrhage with long-term use (at least 1 year) of aspirin as an antiplatelet agent compared to placebo or no treatment showed that the pooled odds ratio for GI bleeding in 65,987 participants was 1.68 (95% CI, 1.51–1.88) [6]. As GI bleeding in survivors of myocardial infarction is independently associated with increased risk of death [adjusted hazard ratio 2.54 (95% CI, 1.66–3.89)] [7] its prevention is of outmost importance. Moreover, it is important to emphasize that chronic use of aspirin is associated not only with gastric complications but also with a variety of lesions in the small bowel, including multiple petechiae, loss of villi, erosions, and round, irregular, or punched-out ulcers [8]. With the aim of decreasing GI toxicity, different formulations of aspirin have been developed, including enteric-coated (EC) aspirin (tablets coated with cellulose, silicon, or other inactive ingredients) [9], buffered aspirin (tablets added with buffering agents) [10], and, more recently, PL2200 (a modified-release lipid-based aspirin) [11]. Among these formulations, EC aspirin has been thoroughly studied in terms of pharmacokinetics (PK) and pharmacodynamics (PD) and is the most widely used formulation for the prevention of arterial thrombotic events. Coating aspirin tablets prevents aspirin absorption in the stomach, thus hypothetically decreasing its GI toxicity, which was mostly attributed to the local effects of the drug. However, clear evidence that EC aspirin is safer than non-EC aspirin (which we will refer to as “plain aspirin” in the rest of the manuscript) in terms of incidence of gastric discomfort and bleeding is lacking. In addition to its dubious advantages in terms of GI safety, it must be emphasized that many reports indicate that EC aspirin is inefficiently absorbed by the intestine in some subjects and, consequently, is unable to inhibit platelet function adequately.

Herein, we will review the PK, pharmacological and clinical efficacy, and GI safety of EC aspirin as well as, when available, other formulations, compared to plain aspirin.

## 2. Pharmacokinetics of Different Aspirin Formulations

Plain aspirin is absorbed in the stomach, where the low pH favors its absorption and protects the active principle from inactivation. EC aspirin, on the other hand, reaches the small intestine, where the higher pH favors drug deacetylation rather than its absorption [12]. A lower bioavailability of EC aspirin compared with plain aspirin can thus be expected. Aspirin is rapidly hydrolyzed to its metabolite salicylic acid by intestinal, plasma, and hepatic esterases [13], and has therefore a systemic bioavailability of only approximately 50% [14], with a C_max_ and an AUC_0–24 h_ that are much lower than those of salicylic acid [15]. After oral administration of 100 mg tablets to healthy subjects, T_max_ is about 0.5 h for plain aspirin [16,17,18] and about 4–5 h for EC aspirin [15,17,18], while C_max_ and AUC are slightly lower with EC aspirin [17,18]. After its absorption, aspirin acetylates platelet COX-1 in the pre-systemic circulation [14], as demonstrated by the fact that inhibition of TxB2 (the stable metabolite of TxA2) production [14] and the appearance of acetylated COX-1 in platelets [15] are detectable before the active principle is measurable in the systemic circulation. Maximal inhibition of TxB2 production in healthy subjects was observed 1–1.5 h after oral dosing with 100 mg plain aspirin [18] and 6–8 h after oral dosing with 100 mg EC aspirin [15,18].

At high doses, buffered aspirin [19,20] and PL2200 [21] displayed PK and PD bioequivalence with plain aspirin, while the bioequivalence of low doses (81–100 mg), which are commonly used in cardiovascular disease (CVD) prophylaxis, has not yet been assessed.

The PK and PD properties of plain aspirin, EC aspirin, buffered aspirin, and PL2200 are summarised in Table 1.

## 3. Pharmacological and Clinical Efficacy of Different Aspirin Formulations

At the beginning of the 21st century, several studies reported a high prevalence of poor pharmacological response to aspirin in treated patients, which was often referred to as “aspirin resistance” [22]. However, a careful analysis of the published studies revealed major flaws in the evaluation of the pharmacological response to aspirin, which was studied using inappropriate and unspecific tests of platelet function [22]. In fact, most attempts to evaluate the efficacy of aspirin using in vivo and in vitro platelet function tests, such as the bleeding time, platelet aggregation assays, and the PFA-100 system, failed to provide consistent data that may be used when discussing the matter of aspirin resistance because of the poor specificity, accuracy, reproducibility, and standardization of the aforementioned tests [22]. The most accurate method to study aspirin resistance is to measure the degree of inhibition of TxA2 formation after drug administration by dosing its stable analogue TxB2 in serum under controlled conditions [23]. The inhibition of at least 95% of serum TxB2 formation has long been considered necessary to prevent thromboxane-dependent platelet activation [24]. Some studies that accurately addressed the issue of aspirin response by measuring serum TxB2 showed inadequate pharmacological inhibition almost exclusively in subjects treated with EC aspirin, as summarized in the following paragraphs.

### 3.1. Studies of Healthy Subjects or Patients on Chronic Treatment for Stable Coronary Artery Disease

In the year 2005, Maree et al. measured serum TxB2 levels in 131 stable coronary artery disease (CAD) patients with a median age of 63 years on chronic low-dose (75 mg o.d.) EC aspirin treatment [25]. In this study population, a suboptimal inhibition of TxB2 formation was found in as many as 44% of the patients. In the same patients, the effects of EC aspirin on platelet aggregation were also studied. Although platelet aggregation tests are less accurate and precise than TxB2 measurement to test the pharmacologic efficacy of aspirin, the authors used arachidonic acid (AA), instead of other platelet agonists as in other studies, which is the specific platelet agonist triggering the COX1/TxA2 pathway of platelet aggregation. As expected, inadequate inhibition of AA-induced platelet aggregation was observed more frequently among patients with high serum TxB2 levels. The in vitro addition of aspirin to patients’ platelet-rich plasma (PRP) samples abolished the residual AA-induced platelet aggregation, thus implying that insufficient bioavailability of aspirin after oral EC aspirin administration was responsible for the inadequate pharmacological response that had been observed in these patients. A very interesting finding of this study was that predictors of poor response to EC aspirin included young age and high body weight. In the following year, the same group of investigators showed that equivalent doses of EC aspirin are less effective than plain aspirin in inhibiting serum TxB2 formation in 71 healthy subjects aged 20 to 50 years [12]. However, in this study, poor pharmacological response to EC aspirin was observed more frequently among subjects with high body weight. The inverse relationship between pharmacological response to EC aspirin and body weight was again confirmed by a study of 148 CAD patients on chronic treatment with 75 mg o.d. EC aspirin for at least three months [26]. Finally, very high percentages of poor responders, defined as <95% inhibition of TxA2 production, were observed among healthy subjects 4 h (39/146, 29%) or 8 h (14/199, 7%) after ingestion of 100 mg EC aspirin, versus none among 40 healthy subjects after ingestion of plain aspirin [27]. Even within the class of EC aspirin, there is variability in the ability to inhibit platelet production of TxA2, as shown by Cox et al., who compared two EC aspirin preparations with plain aspirin: both EC preparations were less effective than plain aspirin in inhibiting TxA2 production, but there was no bioequivalence between the two EC preparations [28].

### 3.2. Studies of Patients Affected by Diseases Associated with Particularly High Cardiovascular Risk

#### 3.2.1. Patients with Diabetes Mellitus

In a randomized, single-blinded, triple-crossover study [29], 40 obese diabetic patients not requiring insulin received three different 325 mg aspirin preparations: plain aspirin, PL2200, and EC aspirin for three days. Aspirin poor responsiveness, defined as <99% inhibition of TxB2 formation in serum at any time during the first 72 h of the study, occurred in a higher proportion of patients receiving the EC preparation (52.8%), compared with plain aspirin (15.8%) or PL2200 (8.1%). Therefore, some degree of aspirin hypo-responsiveness in diabetic patients was observed independently of the aspirin formulation used, although it was much higher in patients treated with EC aspirin. However, it must be noted that the chosen criterion to define aspirin responsiveness in this study was extremely strict (>99% inhibition of TxB2 production), which likely accounts for the high prevalence of “poor responders” also in patients treated with plain aspirin. PK studies, confirming the results of previous reports [16,17,18], showed that T_max_ was significantly lower, while C_max_ and AUC were significantly higher for plain aspirin and PL2200 compared with EC aspirin, suggesting that the observed poor responsiveness to EC aspirin was due to reduced absorption and bioavailability of aspirin. A small study of 42 patients with acute stroke reported that the prevalence of poor pharmacological response to EC aspirin compared to plain aspirin was higher in diabetic patients [30]. In conclusion, plain aspirin should be the preferred formulation for use in diabetic patients.

#### 3.2.2. Patients with Essential Thrombocythemia

Patients with the myeloproliferative neoplasm Essential Thrombocythemia (ET) are at heightened risk for cardiovascular events and, consequently, are prophylactically treated with low-dose aspirin, in analogy with patients with another myeloproliferative neoplasm, Polycythemia Vera, unless their platelet count is >1000 × 10^9^/L, which is associated with high bleeding risk [31]. Several studies reported that these patients may be poor responders to aspirin because the 24 h serum levels of TxB2 were higher than in normal subjects [32]. However, these studies actually tested the recovery of platelet ability to synthesize TxB2 after aspirin administration, rather than the pharmacological response to the drug [31]. In a more recent cross-over study, we showed that poor responsiveness to aspirin is attributable to the use of EC aspirin in these patients [18]. Indeed, our study showed that, in a high proportion of ET patients, serum TxB2 levels are not decreased by 100 mg o.d. EC aspirin, whereas they are adequately suppressed in the same patients by 100 mg o.d. plain aspirin. This difference was attributable to impaired and variable absorption of EC aspirin, with consequent higher T_max_ and lower C_max_ and AUC compared with those of healthy subjects treated with 100 mg o.d. EC aspirin. In contrast, all PK parameters in ET patients were comparable to those of healthy subjects after the oral administration of plain aspirin. In partial agreement with previous reports, we found that the 24 h post-dose serum TxB2 levels were higher in ET patients than in healthy controls, independent of the aspirin formulation used. This difference was attributable to the increased entry of newly formed non-acetylated platelets in the circulation, caused by increased platelet production (which characterizes the disease). Twice daily administration of 100 mg plain aspirin corrected this abnormality in ET patients, suggesting that ET patients with high platelet counts (>400–450 × 10^9^/L) might benefit from 12-h administration of the plain aspirin [18,31].

### 3.3. Studies with Clinical End Points

It is impossible to provide accurate and solid information on the differences between plain aspirin and EC aspirin in preventing cardiovascular events because no direct comparisons between the two formulations have been made in high-quality, large RCTs. However, some indirect evidence exists that EC aspirin could be less effective than plain aspirin.

Rothwell et al., reviewed seven RCTs of low-dose aspirin (75–100 mg o.d.) in the primary prevention of vascular events, which collected data on body weight, height, and individual subject data on baseline characteristics [33]. The most relevant finding of the study was that the ability of 75–100 mg aspirin to reduce cardiovascular events decreased with increasing body weight of the treated subjects: vascular events were reduced by aspirin in subjects weighing 50–69 kg (hazard ratio 0.75 [95% CI 0.65–0.85]) but not in those weighing 70 kg or more (0.95 [0.86–1.04]; 1.09 [0.93–1.29]). The inverse relation between body weight and the efficacy of aspirin was confirmed by the observation that also the increased risk of major bleeding on low-dose aspirin versus control was lost in participants weighing 90 kg or more. Findings were similar in men and women, in people with diabetes, in trials of aspirin in secondary prevention, and in relation to height. Aspirin-mediated reductions in long-term risk of colorectal cancer were also weight-dependent.

Among the seven trials on low-dose aspirin in primary prevention included in Rothwell’s analysis, four employed EC aspirin [34,35,36,37] and one used a delayed-release formulation [38]. The body weight dependence of the effect of low-dose aspirin on cardiovascular events was observed for all formulations, but the loss of effect in participants weighing 70 kg or more was much more evident for EC or delayed-release aspirin [33]. This finding is in perfect agreement with the demonstrations that a poor pharmacological response to EC aspirin is observed more frequently among subjects with high body weight [12,25,26]. Therefore, it is plausible to hypothesize that, given the large prevalence of adult subjects weighing >70 kg who need cardiovascular protection by aspirin, a higher efficacy of aspirin would have been observed if plain aspirin, instead of EC aspirin, had been used for primary (and secondary) prophylaxis of cardiovascular events.

## 4. Gastrointestinal Injury and Bleeding with Different Aspirin Formulations

As already mentioned, aspirin formulations alternative to plain aspirin were developed with the aim of decreasing GI discomfort, mucosal erosions/ulcerations and bleeding that are associated with chronic treatment with plain aspirin. The effective safety advantage of these formulations (EC aspirin in most instances) over plain aspirin was tested in some studies.

### 4.1. Endoscopic Studies in Asymptomatic Healthy Subjects

Some studies tested the effects of the acute administration (5–7 days) of plain aspirin compared with EC aspirin on the prevalence of gastric mucosal erosion and submucosal hemorrhage in healthy asymptomatic subjects who underwent endoscopic examination at the end of treatment (in some studies, endoscopy had also been performed at the beginning of the study, to have a baseline picture of the status of the volunteers). Both formulations of aspirin were given (in a cross-over design for some studies) at doses ranging from 100 mg daily [39], up to 300–325 mg daily [40,41,42,43,44] or even 2.4–3.9 g [40,41,45]. All studies demonstrated that treatment with EC aspirin was associated with a lower prevalence of mucosal injuries, especially when very high doses of aspirin were used, which are commonly administered for the management of inflammatory states rather than for cardiovascular prevention. In none of the studies had episodes of GI bleeding or ulceration been detected. No differences in the frequency of lesions of the gastric mucosa were observed after the oral administration of plain aspirin and buffered aspirin [44].

After a 7-day course of 325 mg aspirin was administered to subjects at high risk of GI complications, endoscopic studies showed that PL2200 was associated with fewer gastric mucosal lesions than plain aspirin [46]. The comparative effects of PL2200 and plain aspirin at low doses and in longer-term studies are necessary to define more accurately the safety profile of PL2200 compared to plain aspirin.

### 4.2. Studies of Upper Gastrointestinal Bleeding or Ulceration in Patients on Chronic Treatment with Aspirin

In the year 1996, a multicenter case-control study by Kelly et al., aimed at assessing aspirin use in the week preceding the acute event or the day of the interview in incident cases of upper GI bleeding (UGIB) and matched controls derived from population census lists [47]. This study investigated the use of plain aspirin, EC aspirin, and buffered aspirin. Data analysis showed that the relative risks (RR) of UGIB for plain, EC, and buffered aspirin preparations at average daily doses of 325 mg or less were 2.6 (95% CI, 1.7–4.0), 2.7 (95% CI, 1.4–5.3) and 3.1 (95% CI, 1.3–7.6), respectively; there were insufficient data to compare the RR of UGIB for doses greater than 325 mg of plain aspirin with those of EC aspirin. The authors concluded that, given the similar RR of major UGIB (both gastric and duodenal) in subjects taking different 325 mg or less aspirin preparations, the systemic effects of the active principle might outweigh the differences in local toxicity, showing no clear benefit in the use of EC preparations. Results mirroring those of Kelly’s study were provided by a population-based case-control study on the risk of upper GI complications (UGIC, bleeding and perforation) associated with the administration of 75–300 mg/day of aspirin [48]. This study used data from the UK-based General Practice Research Database; unlike Kelly’s study, no direct contact was made with patients and controls to better define aspirin exposure, which was solely estimated according to database information. Moreover, only 13% of cases and 7% of controls were exposed to aspirin. Despite these limitations, the RR of UGIC was 2.3 (95% CI, 1.6–3.2) for EC aspirin and 1.9 (95% CI, 1.6–2.3) for plain aspirin, and the results did not change when only patients without antecedents of upper GI disorder were included in the analysis and after adjustment for the use of antiulcer drugs. A Danish population-based cohort study showed similar risks of UGIB in users of low-dose plain aspirin and EC aspirin (standardized incidence rate ratio, 2.6; 95% CI, 1.8–3.5 for plain aspirin vs. 2.6; 95% CI, 2.2–3.0 for EC aspirin) [5]. Only one case-control study on the risk of peptic ulcer bleeding in prophylactic (300 mg daily or less) aspirin users suggested that EC preparations might be safer than other preparations, although no aspirin preparation seemed to be free of the risk of peptic ulcer complications [49].

Garcìa Rodrìguez et al., reviewed the aforementioned four studies and two studies on buffered aspirin published between 1990 and 2001 [50]. The authors calculated a summary RR of serious UGIC (bleeding, perforation, or other serious upper GI events resulting in hospitalization or a visit to a specialist) of 2.6 (95% CI; 2.3, 2.9) for plain aspirin, 5.3 (95% CI; 3.0, 9.2) for buffered aspirin, and 2.4 (95% CI; 1.9, 2.9) for EC aspirin. They therefore concluded that aspirin formulation has little or no effect on the prevention of serious UGIC and hypothesized a likely greater impact of the systemic rather than topical effects of the drug, as suggested by the similar RR of duodenal and gastric lesions. Therefore, the lower incidence of gastric mucosal lesions in endoscopic studies might be explained by the topical effects of the drug, whereas the systemic effects might be predominant in the pathogenesis of UGIC.

The hypothesis about differences between local and systemic toxicity of aspirin is corroborated by evidence from additional studies with somewhat different designs. Some studies showed that the frequency of small bowel mucosal lesions detected by capsule endoscopy was higher in patients taking EC aspirin (which is absorbed in the small intestine) than in those taking non-EC aspirin formulations [51,52,53]. Moreover, although an endoscopic study showed that buffered aspirin formulations reduced the frequency of gastric mucosal erosion compared to plain aspirin [54], the use of buffered aspirin failed to decrease the incidence of peptic ulcer [55].

To summarize, the only source of evidence regarding the decreased GI toxicity of EC aspirin is represented by endoscopic studies, which showed fewer gastric mucosal lesions. However, lesions of the small bowel mucosa appeared to be more frequent with EC aspirin than with non-EC aspirin formulations. These data suggest that GI mucosal lesions are caused by topical effects of aspirin in the region of its absorption. Most importantly, event-driven studies of GI hemorrhage failed to provide data supporting the clinical benefit of EC aspirin or buffered aspirin, therefore suggesting that the systemic effects of the drug, which are unchanged by enteric coating, are to blame for the occurrence of clinically relevant GI complications and bleeding.

## 5. Use of Proton Pump Inhibitors during Chronic Aspirin Treatment

The European Society of Cardiology recommends the use of proton pump inhibitors (PPIs) in patients on chronic aspirin treatment who are at high risk of GI bleeding [56]. PPIs are effective in reducing upper GI clinical events in patients receiving aspirin in the context of dual antiplatelet therapy [57]. The risk, however, is only reduced, not abolished: randomization to PPI therapy reduced 180-day Kaplan-Meier estimates of the primary GI endpoint in low-dose aspirin recipients to 1.2% from 3.1% [57]. These results are in keeping with a previous literature review focused on PPIs effectiveness in patients taking aspirin as single antiplatelet therapy [58] and with the results of a Swedish cohort study [59], which highlighted that compliance to continuous use of PPIs was pivotal, as intermittent use was associated with increased risk of adverse GI outcomes and of aspirin discontinuation. As an alternative to PPIs, histamine H2 receptor antagonists (H2RAs) can be used, although they have been proven less effective than PPIs in the prevention of GI complications in patients on low dose aspirin alone [60] or in combination with anti-P2Y12 drugs [61].

## 6. Conclusions

**Figure 1:**
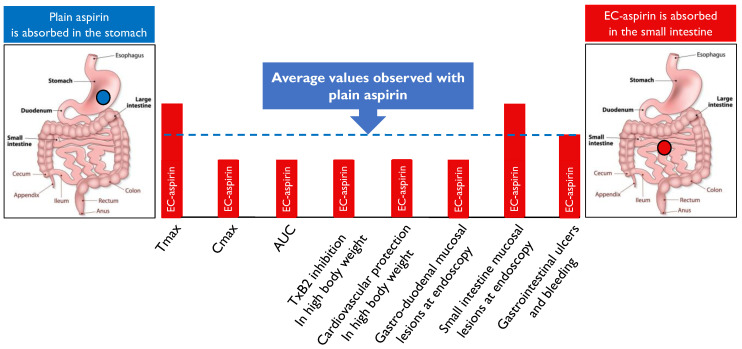
Pharmacological profile, clinical efficacy, and safety of enteric coated (EC) aspirin compared to plain aspirin. The height of the histograms shown in the figure is not reflecting real data and should be interpreted as illustrative of the average values obtained in several studies with EC-aspirin relative to plain aspirin (higher, equal, lower). Pharmacokinetic parameters (usually measured in serum): Tmax = time to peak drug concentration; Cmax = peak drug concentration; AUC = Area Under the Curve (integral of drug concentration as a function of time). TxB2 = thromboxane B2 (a stable metabolite of thromboxane A2).

The absorption of EC aspirin is delayed and erratic, resulting in less effective inhibition of the platelet production of TxA2, thus providing less effective inhibition of platelet function, especially in subjects with high body weight. Such inferiority in pharmacological efficacy seems to have a clinical impact, as shown by a meta-analysis of RCTs, predominantly on primary cardiovascular prevention, which revealed that lack of protection by low-dose aspirin in subjects weighing >70 kg was particularly evident in subjects treated with EC aspirin. On the other hand, there is no evidence that EC aspirin protects from clinically relevant GI bleeding and ulceration. Differences in the incidence of asymptomatic lesions of the GI mucosa detected by endoscopy reflect the effects of the drug on the site of its absorption: more lesions of the gastric mucosa can be observed after plain aspirin administration, while more lesions of the small bowel are observed after EC aspirin ingestion (the main differences between plain aspirin and EC aspirin are summarized in Figure 1).

The use of PPIs is recommended for patients on chronic aspirin with risk factors for GI bleeding, which include a history of peptic ulcer disease or gastrointestinal bleeding, older age, concomitant use of NSAIDs, concomitant use of anticoagulants or other platelet aggregation inhibitors, and the presence of severe co-morbidities [62]. Coformulations of aspirin and PPIs could be considered for patients for whom polypharmacy and poor compliance are a reason for concern. H2RAs can be considered as alternatives when PPIs are unavailable or contraindicated.

Considering its more favorable pharmacological profile, plain aspirin should be the preferred formulation for cardiovascular prevention. The improvement in P2Y12 inhibition obtained with the newer antiplatelet drugs prasugrel and ticagrelor, which have a more efficient PK than clopidogrel [63] could be replicated for COX-1 inhibition by using an older antiplatelet drug with a more efficient PK than EC aspirin, which is still the most widely used aspirin formulation in the setting of cardiovascular prevention.

## Figures and Tables

**Table 1:** Pharmacokinetics and pharmacodynamics of plain aspirin, enteric-coated aspirin, buffered aspirin, and PL2200 after oral administration to healthy subjects.

	Plain Aspirin (100 mg Tablets)	EC Aspirin (100 mg Tablets)	Buffered Aspirin (325 mg Tablets)	PL2200 (325 mg Tablets)
Preparations	Uncoated tablets	Tablets coated with inactive ingredients	Aspirin associated with buffering agents *	Complex of aspirin and lipidic excipients
Site of absorption	Stomach	Small intestine	Stomach	Duodenum
Time to maximal plasma concentration of aspirin	0.5 h	4 h	0.4 h	1 h
Time to maximal inhibition of thromboxane B2 production	1–1.5 h	6–8 h	1 h	2 h

* Calcium carbonate, magnesium oxide, magnesium carbonate; abbreviations: EC, enteric coated.

## Data Availability

Not applicable.
